# A Geometry-Based Welding Distortion Prediction Tool

**DOI:** 10.3390/ma14174789

**Published:** 2021-08-24

**Authors:** Ignacio Granell, Abel Ramos, Alberto Carnicero

**Affiliations:** 1 Institute for Research in Technology, Pontifical Comillas University, Santa Cruz de Marcenado 26, 28015 Madrid, Spain; igranellh@gmail.com; 2 Ansys Inc., Paseo de la Castellana, 81, Planta 9, 28046 Madrid, Spain; abel.ramos@ansys.com

**Keywords:** Ansys, welding distortion, finite element method, thermoelastoplastic method, welding simulation

## Abstract

The prediction of welding distortion requires expertise in computer simulation programs, a clear definition of the nonlinear material properties, and mesh settings together with the nonlinear solution settings of a coupled thermal–structural analysis. The purpose of this paper is to present the validation of an automatic simulation tool implemented in Ansys using Python scripting. This tool allows users to automate the preparation of the simulation model with a reduced number of inputs. The goal was, based on some assumptions, to provide an automated simulation setup that enables users to predict accurate distortion during the welding manufacturing process. Any geometry prepared in a CAD software can be used as the input, which gave us much geometrical flexibility in the shapes and sizes to be modeled. A thermomechanical loosely coupled analysis approach together with element birth and death technology was used to predict the distortions. The automation of the setup enables both simulation and manufacturing engineers to perform welding-induced distortion prediction. The results showed that the method proposed predicts distortion with 80–98% accuracy.

## 1. Introduction

Welding distortion is defined as the distortion caused by the nonuniform expansion and contraction of the weld and base metal during the heating and cooling cycle produced by a welding process. This is an undesirable and mostly inevitable permanent change in shape, which can usually be managed by constraining the parts or changing the welding process.

These welding-induced distortions may affect the safety, functionality, and durability of a structure. Therefore, it is very important to take them into consideration in the engineering design process and minimize their occurrence. Predicting the change of the shape of a component allows designers to modify their products or manufacturing processes to achieve the level of performance or quality required. Traditionally, trial and error tests were used to solve these problems. Currently, digital tools involving finite element simulations and databases can greatly reduce the calculation time and increase the number of possible numerical tests, as well as the accuracy of the results [1].

However, most numerical simulation tools require expert knowledge, not only of the welding process, but also of the numerical methods implemented in those tools. Finite element simulation of the welding process has been successfully used to predict temperatures, strains, or displacements and stresses. An exhaustive review of the accuracy of different approaches using finite element methods can be found in [2].

Over the last thirty years, welding distortion has been amply researched, and several computational welding mechanics methods have been developed. The two most frequently used methods for computational welding simulation are the thermoelastoplastic method and the inherent strain method, which have been combined by many researchers to develop their own methods.

The thermoelastoplastic method (TEP) was introduced originally by Ueda and Yamakawa [3] to study the thermodynamic process in welding structures. It involves a coupled thermal and mechanical FE simulation. Regarding this method, Long et al. [4] used the TEP in the prediction of distortion in butt welds. Teng et al. [5] analyzed residual stresses and distortions in T-joint welds. Schenk et al. [6] used it to model buckling distortion, and many other researchers have used this method in the prediction of welding distortion, even including the change of phase in the material model [7]. As some simulations mentioned require a long computational time, the simulation sometimes requires approaches such as substructuring [8] or block-dumping [9].

The inherent strain method (ISM), introduced by Ueda et al. in 1975 [10], employs a different approach, using inherent strain to quantify residual stresses in long welds. ISM is usually performed by an elastic finite element simulation, and it is typically used in the prediction of large, welded structures [11] and sometimes in combination with the TEP method [12,13,14]. Souloumiac et al. [15] introduced a local–global approach. Plastic strains are computed from a local 3D model by the TEP, applying those strains as initial strains in the elastic calculation of the entire structure.

In 2007, Deng et al. [16] developed the inherent deformation method (IDM). This nonlocal alternative to ISM is frequently applied in the study of large welding structures using linear material models (see [17,18]). In 2012, Khurram et al. [19] investigated an efficient FE technique using an equivalent force method based on shrinkage to calculate deformations and residual stresses in butt joints.

The characterization of the welding process involves many parameters. Rong et al. [20] provided a detailed summary of the research status on welding distortion and residual stresses’ prediction by FE analysis. Research conducted in this field has confirmed that expert knowledge of FE theory and numerical simulation are needed to simulate a welding process, even when using a commercial FE code. As a result, they are thus generally unavailable for welding or engineering professionals who lack this expertise. This is the main reason for the creation of an automatic methodology and its implementation as a user-friendly tool using the Ansys customization toolkit. The tool automates the setup creation for these simulations, allowing professionals with advanced welding expertise but less experience in finite element simulations to address these complex analyses.

The methodology proposed in the tool, and described in this paper, uses a TEP analysis combined with the birth and death FE technique to simulate the incremental deposition of filler material during the welding process. The birth and death technique was previously used to predict residual stress and distortion in flat-bar-stiffened plates (Gannom et al. [21]), to simulate laser welding and TIG procedures with Ansys (Capriccioli et al. [22]), and in the analysis of butt-welded plates (Chen et al. [23]). The novelty of the methodology presented in this paper lies not in the purposes for which it is applied, but in the automation of the setup process and its validation through four comparisons with the experimental results. The main goal was to enable simulation users to prepare welding models in a short period of time to predict welding bending and angular distortion.

The remainder of the paper is organized as follows. Section 2 describes the methodology, the development of the welding tool, and the hypotheses on which this methodology is based. Section 3 is focused on the presentation of the welding-induced distortion prediction results. Four experimental cases are compared with the numerical simulations using the tool developed. Finally, Section 4 summarizes the main conclusions of this study.

## 2. Materials and Methods

As mentioned previously, the preparation of a model to simulate a welding process requires good knowledge, not only of welding procedures, but also the numerical simulation and software handing skills. Moreover, the definition of the model and its solution comprise an extremely laborious process as this requires many steps.

The manual steps that a user must take in Ansys to prepare the setup of the model implies the following:

- The preparation of the geometry;
- The creation of all the simulation load steps that will be used to simulate both transient and structural analysis. Each simulation setup needs to be split into several steps to model the transient process, and each of these individual simulation steps is called the “load step”;
- Calculations and model setup of the proper timings depending on the welding speed for both analyses. Each of the simulation load steps has its own timing depending on the process parameters;
- The selection of the groups of elements from the deposition bodies. Each geometry part denominated as “deposition body” is meshed with a certain number of elements, which must be identified for further processing;
- Manual transient heat input during the welding process in the transient thermal analysis. This input needs to be modeled as a moving load over time through the geometry;
- Element deactivation and activation logic during the welding process. These steps are dependent on the geometry, the number of welding toes, the welding setup, and the solving time. These steps apply to both thermal and structural analyses;
- The setup of the deposition bodies’ reference temperatures;
- The creation of cooling load steps after the welding process is finished.

To streamline these manual steps, the procedure presented in this paper was integrated in a CAE tool, allowing users to get rid of most of the manual solver settings.

**Figure 1:**
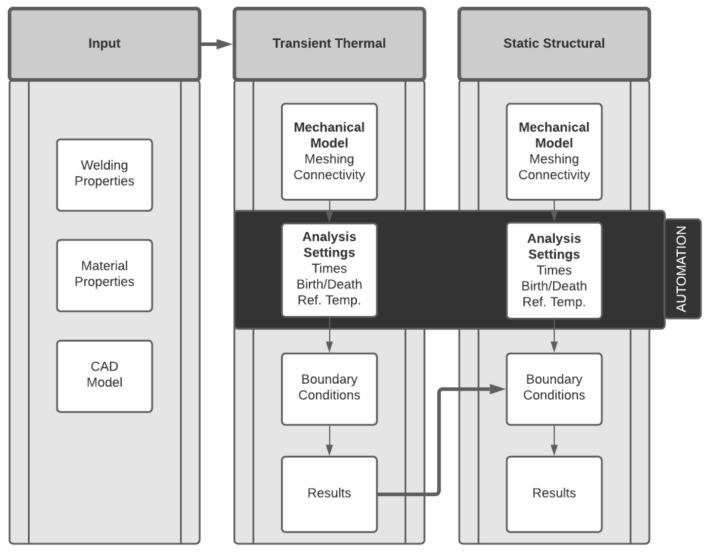
Proposed methodology workflow.

Figure 1 shows the workflow for the simulation process that was employed to predict the welding distortion. This paper explains the method used to automate the key analysis setup steps, taking as a starting point the CAD geometry of the parts, which is easier to handle than a finite element model.

The methodology implemented starts with the assumption that the welding settings, material properties, and CAD model are known. This information is used as the input data for modeling the welding process, which is divided into two steps, a transient thermal analysis loosely coupled to a static structural analysis.

### 2.1. CAD Geometry

**Figure 2:**
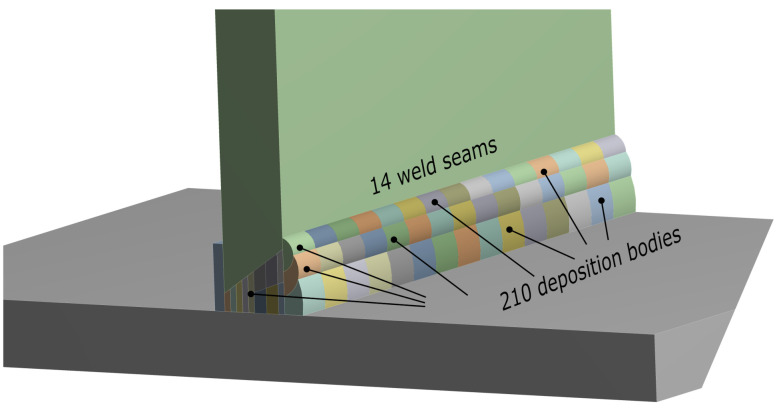
Prepared CAD geometry. Welding seams are modeled and split into different deposition bodies.

The components to be welded are modeled in a CAD system. The actual representation and shape of the welding seams are modeled in this initial geometry. Its preparation implies the division of the welding seams into multiple geometric bodies. This operation is available in all the commercial CAD software and allows the user to model any geometry of the welding seam using split operations. This way of working enables the user to concentrate on the geometry model, which is easier to handle with modern CAD systems. Each one of those divisions is referred to in this paper as “deposition bodies”. The finite elements attached to these deposition bodies are activated automatically, load step by load step, using the element birth and death technique to simulate the progressive process of filler material deposition. This technique supports the identification, automation, and activation of each load step of the key mesh elements to model the welding process with filling material. This approach, based on the initial geometry, allows the user to create any shape needed to model all types of welding seams, having no restrictions in modeling welding lengths and/or continued or split welding seams. As an example, Figure 2 shows a properly prepared geometry ready for simulation. As can be seen, each welding seam is sliced into several deposition bodies. The methodology presented uses these geometric bodies to automatically configure the settings of the simulation, and therefore, it is a methodology based on the geometry and not on the mesh, as are the most-used methods to predict welding distortion. The fact that the methodology is geometry-based offers limitless possibilities for modeling complex geometries. In short, the user must divide the welding seams into several deposition bodies in the input CAD geometry. The larger the number of deposition bodies, the longer the computational time and the better the precision will be. This point is further developed in Section 2.4

### 2.2. Welding Setup

The welding process needs to be set up properly to perform a simulation. To make the input data as simple as possible, only three parameters are required: welding time, cooling time, and reference temperature. Welding time is the time spent in the fabrication of the weld joint. Welding time combined with the number of deposition bodies modeled in the CAD software allows the system to compute the time steps of the solution process. Cooling time is the time spent cooling the part down to room temperature. In the cases presented in this paper, this cooling time had no influence on the final deformation obtained. This statement implies that increasing or reducing either the convection film coefficient or the cooling total time during this last phase has no impact on the welding-induced distortion prediction. This condition only applies if the materials used are temperature-dependent rather than time-dependent. If the material models include a change of phase or time-dependent material properties, this time would have a significant impact on the results. This hypothesis allowed the authors to increase the convection film coefficient during the cooling phase of each simulation to accelerate them and, therefore, reduce the total computational time. Finally, the reference temperature is the temperature at which bodies are deposited in the welding pool. From a physical point of view, that temperature would be the melting temperature. It was assumed that the elements created at this reference temperature have no initial strain (Equation (1)), and their shrinkage will occur while the element temperature drops depending on the thermal boundary conditions.

(1) $$\varepsilon =\alpha \left(T-T_{ref}\right)$$

### 2.3. Finite Element Analysis

The methodology used to predict welding distortion uses the finite element method (FEM) to perform a loosely coupled TEP analysis. This simulation technique allowed the authors to use the transient temperature results from a thermal analysis as a body loads into a static structural analysis. As was pointed out in the Introduction, this loosely coupled analysis has been used by many researchers due to the thermal process having a decisive effect on the mechanical process, but the mechanical process has a small influence on the thermal one. This analysis considers the contribution of the transient temperature field to stress through the thermal expansion coefficient, as well as the temperature-dependent thermal–physical and mechanical material properties.

**Figure 3:**
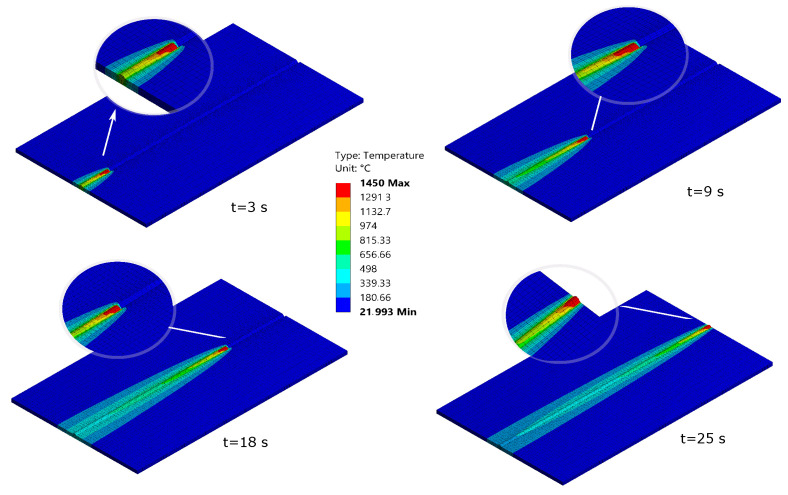
Element birth process in the thermal analysis of a welding process of the IN718 material [24].

Transient thermal analysis was used to compute the thermal field and its evolution over time. The mesh element birth and death technique were used to reproduce the welding filler deposition process, which was modeled by several deposition bodies created previously with a CAD software. Element birth and death allow users to activate/deactivate elements of a mesh under a physical condition. On the one hand, elements will be born, a the welding pool, at the reference temperature and at the welding speed. On the other hand, that temperature is associated with an initial state of no strain. The elements are left to cool, and when the welding process is finished (i.e., all the thermal finite elements are alive in the model), a cooling step is applied to allow the complete structure to evolve and stabilize its temperature to room temperature. Figure 3 shows four different instants of the welding process. Zoomed areas depict the finite element being born automatically during the welding process.

The thermal analysis uses the 3D thermal conduction element Solid70. This element has eight nodes with a single degree of freedom, temperature, at each node, and therefore uses linear interpolation.

There were two thermal boundary conditions applied in this analysis: convection at room temperature on all the external surfaces of the geometry; an internal transient heat generated in the welding pool. This heat was introduced into the model by means of the application of the reference temperature in the element birth formulation. Therefore, this is not actually an internal heat generation, but the deposition (birth) of a group of finite elements (modeling a deposition body) at the reference temperature.

The methodology proposed automates the creation of one deposition body in each load step. Time is automatically calculated to set up the analysis settings for the transient thermal simulation. The time step is calculated according to the number of deposition bodies in the CAD geometry and the total welding time, which can be obtained by the welding speed and the total length. The calculated time for each time step, $t_{i}$, is:

(2) $$t_{i}=\left(i-1\right)\cdot \frac{Welding\;time}{Number\;bodies}$$

Automatic substepping capabilities in Ansys were used to allow the solver to choose the best time increment to be used in each solution iteration, depending on the model difficulty.

The user has to define the reference temperature that is used to model the heat input during the manufacturing simulation. This temperature is automatically applied to the elements activated for each deposition body. It is maintained during the load step and is left to cool when the next deposition body is deposited. Once the thermal field is computed, the structural analysis is performed.

Static structural analysis with active large deflection was used to calculate the geometrical distortions. The structural boundary conditions applied on each case study depend on how the manufacturing process is carried out. The degrees of freedom of the element’s nodes are restricted in the regions where clamping devices are used to fix the specimen during welding. This analysis used the 3D linear element Solid185, although it is possible to use the quadratic version of it (Solid186). The analysis settings for the static structural simulations replicated the times used in each load step from the thermal analysis.

**Figure 4:**
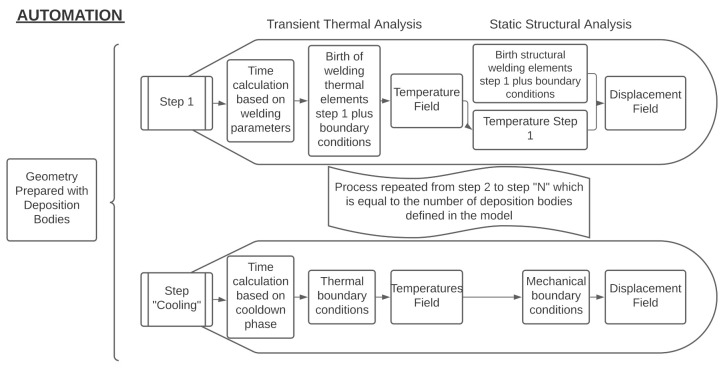
Automatic FE analysis setup.

Figure 4 shows a representation of this automatic process, which is managed by the user as a “black-box”.

To model the final cooling phase of the process, an extra final step is added with no heat applied and no new bodies being activated.

All the steps described in this section are needed to prepare the analysis setup, regardless of the FEM tool used (Figure 1). The automation was developed to ease the effort of preparing the most time-consuming parts of the workflow, as described in Figure 4. Several commercial FEM tools allow the user to customize workflows by interacting with the interface using some coding. In this case, the Ansys Application Customization Toolkit (ACT) was chosen to automate this process because it enables engineers to automate routine workflows using Python scripting. The Python code was structured into four sections:

- Input: the routine that obtains all the user inputs from the interface;
- The identification of deposition bodies: the routine that identifies the different deposition bodies in the correct order to prepare the right setup of each analysis and for each body as described in Section 2.1;
- Transient thermal: the routine that prepares the transient analysis based on the user input and deposition bodies as described in Section 2.3;
- Static structural: the routine that prepares the structural analysis based on the user input, the deposition bodies’ information, and the thermal results as described on Section 2.3.

#### Material Properties

It is mandatory to define the thermal material properties for both the base and filler materials to be able to perform the thermal simulation. These properties are density, thermal conductivity, and heat capacity. Radiation could be added to the model if necessary, regarding the boundary conditions of the simulated experiment. In all the analyses reported in this paper, radiation was not considered. For the static structural analysis, the material properties required are: thermal expansion coefficient, Young’s modulus, Poisson’s ratio, yield stress, and tangent modulus. All these properties are defined as temperature-dependent. Improved models could be used to model the material behavior. To check the less precise cases, the authors used as few parameters as possible to define the material model.

### 2.4. Hypotheses

**Figure 5:**
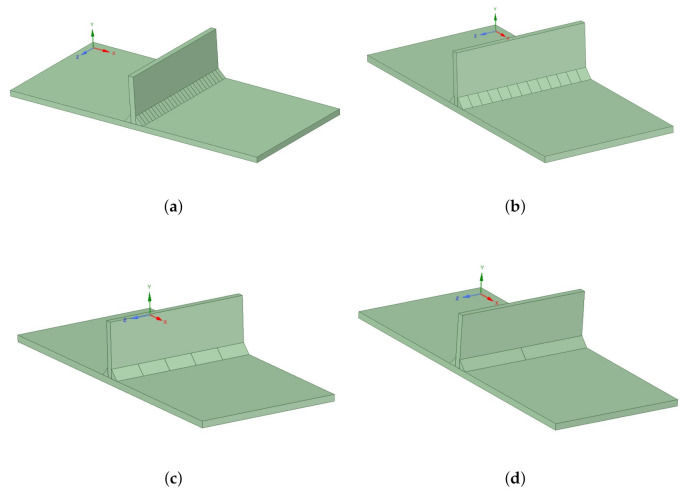
Analyzed geometries: (**a**) 52 divisions, (**b**) 26 divisions, (**c**) 10 divisions, and (**d**) 4 divisions. This geometry was used with S355, S700, and S960 steel to model the case defined by Bhatti et al. [25].

In this section, the different hypotheses formulated before the validation of the presented methodology are stated. All the simulations performed to obtain the results presented in the next section assumed these hypotheses, and we used them systematically in every case:

- Reference temperature. As was pointed out previously, this temperature is defined as the temperature at which a structure would suffer no strain under the application of a load.
  This point was selected as the temperature value at which the tangent Young’s modulus reaches a minimum value, and its constant as the temperature keeps growing. From a practical point of view, the authors took around 80–90% of the melting temperature;
- Hardening property. Values were selected in the range of 0.5% and 2% of the Young’s modulus following the recommendations by Bhatti et al. [25]. It is important to state that not all researchers give importance to this material property;
- Length of the deposition bodies. This is one of the critical points of the implemented method because there are no previous studies about it. A parametric analysis was performed to address the effect of the ratio “deposition body length versus total weld seam length”. This analysis allowed the authors to define some guidelines on the maximum length to be used in the deposition bodies.
  The recommendations in this paper were the results of a sensitivity analysis performed using the experimental cases reported by Bhatti et al. [25] with three different steels. They were also validated with the four cases presented in Section 3. In that paper, a T-joint with two weld beads was tested using three different materials. In each of them, the welding bead geometry was divided into 2, 5, 13, and 26 bodies. Hence, body lengths of 65 mm (50%), 26 mm (20%), 10 mm (≈8%), and 5 mm (≈4%) were analyzed. Figure 5 shows the four CAD geometries.

**Figure 6:**
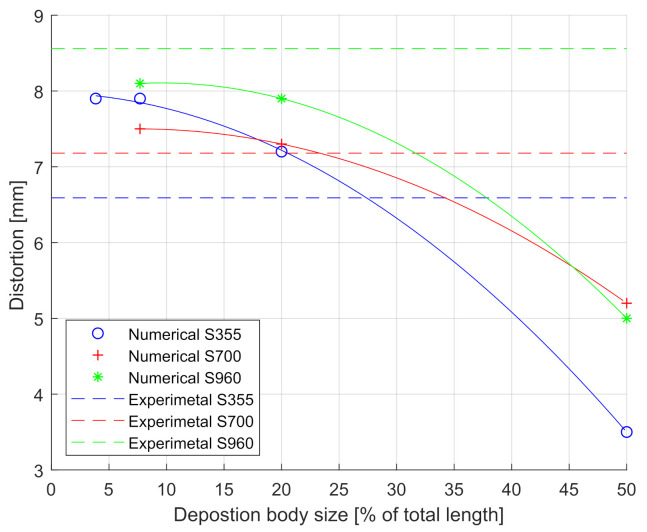
Sensitivity analysis of body deposition size. Experimental results from Bhatti et al. [25].

The results obtained in the three cases (S355, S700, and S960) are shown in Figure 6. This figure shows the h-convergence of the mesh. The X-axis plots the ratio “deposition body length versus weld seam total length”, whereas the Y-axis plots the distortion in millimeters. One single distortion result is shown in Figure 6 for each specimen’s experiment, depicted as a straight line. Simulation distortion predictions using different body lengths are also shown together with the experimental results, to compare and study the effect of the body length on the predicted distortion. The case of a deposition body size of ≈4% of the total length gave a low difference with respect to the case of the 26-deposition-body model (≈8%) results. The large amount of computational time spent in this simulation compared to the previous one did not seem to compensate for the reduction in body size by looking at the gain in precision achieved. However, it is up to the user to decide the tradeoff between the deposition body length (mesh size) and the computational time. An upper bound of 10% should ensure enough accuracy, considering the results shown on the h-convergence of the mesh.

## 3. Results

In this section, the results of the research are presented. The objective was to prove the validity of this implementation in predicting welding-induced angular and bending distortions. The validation was performed by simulating the experimental results fully reported by four research groups. These experimental results were chosen to provide a variety of geometries, materials, and welding processes. The validation cases were:

- A T-shaped joint fabricated in Aluminum EN-AW-6082-T6 [26];
- IN718 superalloy plate TIG welding [24];
- A double-welded T-joint fabricated in three different steels [25];
- A multipass fillet welded tube-to-pipe T-joint [27].

### 3.1. T-Shaped Joint Fabricated in Aluminum EN-AW-6082-T6

Renzi et al.’s [26] specimen consisted of a T-shaped joint fabricated in Aluminum EN-AW-6082-T6. This alloy is a high-strength alloy for highly loaded structural applications and has good resistance to dynamic loading conditions. Typical applications are scaffolding elements, machine building, mobile cranes, rail coach parts, offshore constructions, and lightweight manufacturing in the railway and automotive industries.

The length of the welding bead was 130 mm, and the deposition bodies had a length of 5 mm (3.8% of the total length); therefore, 26 deposition bodies were introduced. The resultant mesh created with a hex-dominant method and an element size of 5 mm had 2483 nodes and 1309 elements. The welding speed was 10 mm/s; therefore, the welding time was 13 s, resulting in 26 load steps with a duration of 0.5 s each. The reference temperature was 450 ${}^{\circ }$C. In the thermal analysis, convection was applied to the external faces of the model. The convection coefficient value was 10 W/m${}^{2}$K during the welding phase. Renzi et al. [26] specified that during the welding phase, the parts were clamped, constraining the two upper corners of the vertical plate and two other corners of the horizontal plate. Nevertheless, during the cooling phase, the part was released and left to evolve freely. The structural boundary conditions used to reproduce this situation were a six-degree-of-freedom-constrained remote displacement in the mentioned corners during the first 26 load steps (welding phase) and an equally constrained remote displacement in the upper edge of the vertical plate during the cooling. During the whole simulation, a compression-only support was applied on the lower face of the horizontal plate to reproduce the effect of the welding table.

**Figure 7:**
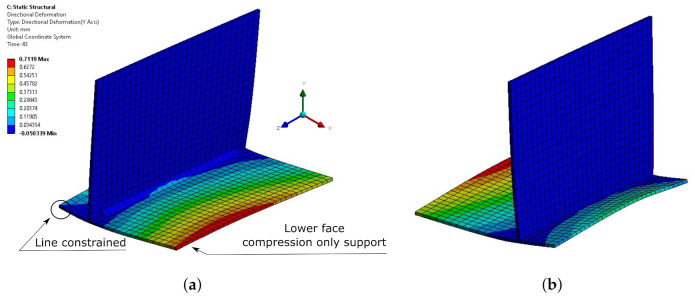
Boundary conditions and vertical deformation contours for the Renzi model (15 scale factors): (**a**) frontal view; (**b**) rear view.

**Figure 8:**
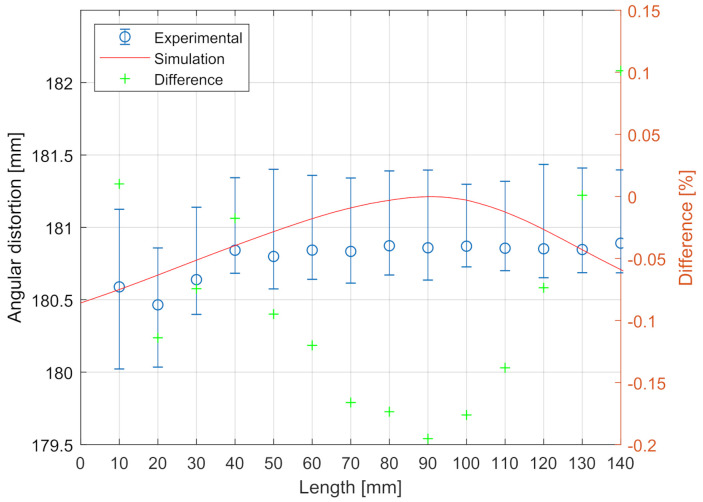
Predicted results against experimental results.

The deformations contour obtained in the simulation is shown in Figure 7. This distortion obtained using the proposed methodology was compared with the experimental distortion values obtained by the authors. Figure 8 contains the predicted angular distortion along the welding seam measured in the edges of the plate and the experimental values reported in [26]. As can be observed, the results matched the experimental distortion at all the measured points along the whole length of the T-shaped joint. The predicted angular distortion was inside the experimental variability for all the specimens manufactured. The maximum difference observed between the angular distortion predicted and measured was less than −0.2%.

### 3.2. IN718 Superalloy Plate TIG Welding

Dye et al.’s [24] specimen was a 200 × 100 × 2 mm IN718 plate. This superalloy is used in applications where high-performance mechanical properties are required at very high temperatures. IN718 is ideal for use in turbines in supercritical power plants, jet engines, and high-speed airframe parts.

In this case, the welding was not used as a joint. The weld bead was deposited in the midsection of the plate, causing it to bend and distort.

The length of the welding seam was 180 mm, and the eighteen deposition bodies had a length of 10 mm (5.5% of the total length). The welding speed was 1.59 mm/s, so the total welding time was 113.2 s. The reference temperature used was 1150 ${}^{\circ }$C. The mesh created with a hex-dominant method and an element size of 2 mm had 10671 nodes and 5721 elements. In the thermal analysis, the convection coefficient was 35 W/m${}^{2}$k, as was referenced in [24]. No boundary conditions or information about the way the parts were supported was provided in [24]. Based on experience and the photographs available, a six-degree-of-freedom-constrained remote displacement was applied on both short edges of the plate.

**Figure 9:**
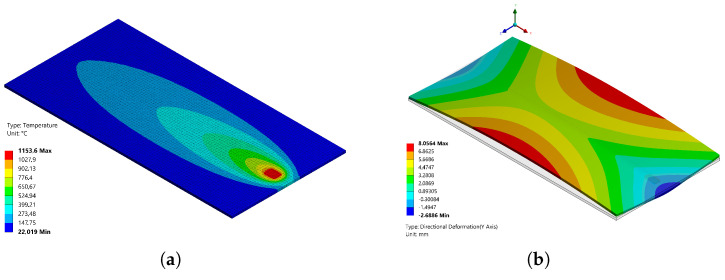
(**a**) Temperature (${}^{\circ }$C) and (**b**) boundary conditions and vertical displacement (mm) contours in the Dye model.

Figure 9 shows the predicted vertical deformation contours of the model.

**Figure 10:**
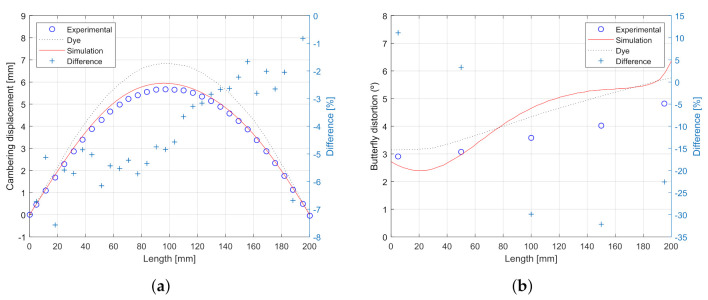
Predicted vs. experimental: (**a**) cambering distortion and (**b**) butterfly distortion.

In the original paper, a distinction between “cambering” or bending distortion and “butterfly” or angular distortion was made. Figure 10 compares the corresponding predicted distortion against the experimental values.

As depicted in Figure 9, cambering distortion showed the sheet deformation along the longitudinal axis of the welding seam, showing the maximum displacement approximately at the middle of the plate. On the other side, butterfly distortion plots the angle created in the perpendicular direction from the welding seam. The minimum angle was obtained close to the welding seam, whereas the maximum was shown in the extremes of the metal sheet.

Bending distortion results showed a great agreement between the prediction and measurements. The comparison showed a maximum difference of less than 8% between the measurements on the specimens and the simulation along all the lengths of it.

Whereas the difference predicted in the angular distortion was a bit bigger in this case (less than 30%), it is important to remark that it is not exactly clear how this measurement was performed on the specimen, so the authors used their best engineering judgment to address these values to compare the simulation results with the measurements reported.

### 3.3. A Double-Welded T-Joint Fabricated in Three Different Steels

The specimen that Bhatti et al. [25] reported is a double-welded T-joint fabricated in three different steels (S355, S700, and S960).

This model was previously used as a case to perform the sensitivity analysis. In this case, 13 deposition bodies of 10 mm (7.7% of the total length) were used. The mesh created with a hex-dominant method and an element size of 5 mm had 3380 nodes and 3276 elements. The welding speed was 8.3 mm/s, and the welding time was 16.65 s. The reference temperature was 800, 900, and 1020 ${}^{\circ }$C for S355, S700, and S960, respectively. The convection coefficient value was 20 W/m${}^{2}$K during the welding phase. For the structural analysis and based on the description of the welding procedure, one of the horizontal edges of the plates was fully constrained.

**Figure 11:**
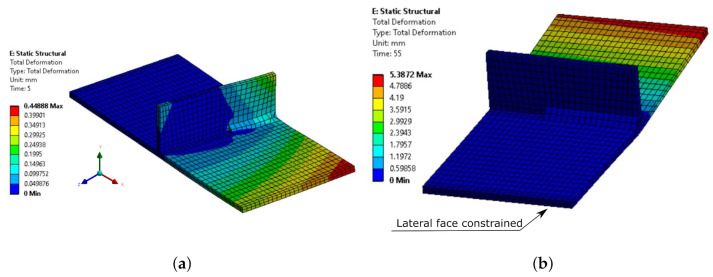
Vertical deformation contours for the Bhatti model at (**a**) 5 s (41 scale factors) and (**b**) 55 s (3 scale factors), as well as the boundary conditions.

Figure 11 shows the contours of the S700 model at two different instants of the simulation.

Table 1 details the predicted values and the error obtained in the simulation. The main experimental result compared in this scenario was the maximum vertical displacement. The maximum difference obtained at the end of the simulation was always lower than 20% for all the materials.

### 3.4. A Multipass Fillet Welded Tube-to-Pipe T-Joints

Vetriselvan et al. [27] reported a multipass tube-to-pipe joint. The material used here was EN-S355J2G3 steel. This weldable high-strength structural steel has a wide range of structural applications: freight cars, transmission towers, cranes, pipes, building structures, power plants, oil and gas equipment, machinery, etc.

The joint was a circumferential weld composed of three welding seams of approximately 44.5π mm in length. Each seam was divided into eight deposition bodies (12.5% of the total length). According to [27], the welding speed was 2 mm/s, with a total welding time of 420 s. Nevertheless, the graphics shown in [27] gave a time of 800 s. As the objective was to validate the proposed methodology against the experimental measurements, a welding time of 800 s was used. The reference temperature was taken as 1300 ${}^{\circ }$C. The mesh created with a hex-dominant method and an element size of 6 mm had 6931 nodes and 6810 elements. In the thermal analysis, the convection coefficient was 20 W/m${}^{2}$k. No boundary conditions or information about the way the parts were supported was provided in [27], but based on experience and the photographs available, a six-degree-of-freedom-constrained remote displacement was applied to the bottom edge of the pipe.

**Figure 12:**
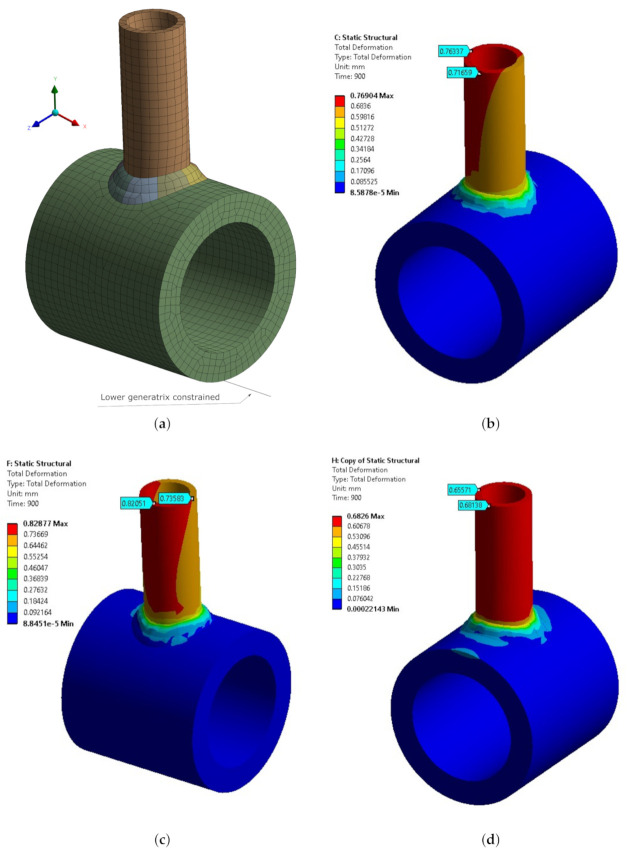
(**a**) Finite Element model and boundary conditions and predicted distortion contours at steady state for Sequences 1 (**b**), 2 (**c**), and 3 (**d**) in the Vetriselvan model.

Three different welding sequences were studied, and as the experimental values were provided for the three of them, they were reproduced in this paper as well. Figure 12 shows the deformation contours after cooling was performed on the three sequence models. Table 2 compares the predicted distortions and the experimental values reported by Vetriselvan et al. [27].

In this test case, two measurements were compared, deformation in the X-axis and in the Z-axis. The maximum difference obtained was 35% in the first welding sequence along the X-direction. However, the average value for all differences considering all welding sequences in both directions was 13.7%, and the most accurate sequence was achieved in the third specimen with a maximum error of 5.4%.

## 4. Discussion and Conclusions

Table 3 summarizes the results obtained in the four cases simulated, showing the biggest difference between the numerical solution and the empirical measurements after the welding process. The results presented in the table display that the automation of the thermoelastoplastic methodology using the birth and death FEM technique in Ansys was valid.

Despite the variety of geometries, materials, and empirical measurements of the tested cases, the difference was lower than 20% in most cases. Therefore, the prediction using this approach (defined as the difference between the simulation result vs. the experimental measurements) remained in the range of 98% to 80% accuracy in most of the cases. The inaccuracies were, in the eyes of the authors, caused by one, or a combination, of the following possible facts:

- A low accuracy in the material models due to a lack of properties and the low resolution of the source graphics;
- A possible coarse mesh, with a finer mesh, would probably provide more accurate results;
- The size of the deposition bodies. As has been proven before, convergence increases with smaller deposition bodies;
- A possible misunderstanding regarding the measurement method used by the paper’s authors. This could happen especially in the cases of Vetriselvan [27] and Dye [24];
- A low resolution in the experimental values’ source graphics;
- All the experimental results provided (except for [26]) reported one single experiment value, without reporting any variability of the experiment itself and the measurement performed.

It is important to highlight the fact that these results were obtained without an exhaustive preparation of the model; therefore, more accurate estimations (and longer computational times) could be achieved with further work in the finite element model, the material properties’ definition, etc. The goal of this preparation of the model was to test the validity of the tool results under the condition of nonexpert users. Based on the results, the authors recommend using a deposition body length lower than 10% of the total welding length to obtain reasonable results.

The novelty of the purposed method is the full automation, using Python in the Ansys environment, of the simulation analysis setup. This automation is available as an Ansys app, to help the user simplify the preparation of welding models for simulation. This is an important contribution from a technological point of view because it implements, in a well-known FEM code, a specific tool that enables its users to speed up the setup to simulate welding processes.

Finally, as future work, it is possible to apply this methodology to simulate additive manufacturing processes as direct energy deposition techniques. The methodology can also be updated to simulate laser welding or by improved with new welding parameter inputs, based not only on the initial temperature, but also on the energy.

## Figures and Tables

**Table 1:** Bhatti’s analysis.

Steel	Experimental (mm)	Predicted (mm)	Difference (%)
S355	6.59	7.77	18.1
S700	7.18	7.46	3.9
S960	8.86	8.07	8.7

**Table 2:** Vetriselvan’s analysis.

Weld Sequence	Experimental	Predicted	Difference
(Direction)	(mm)	(mm)	(%)
1-x	1.10	0.72	34.9
1-z	1.0	0.76	23.7
2-x	0.72	0.74	2.2
2-z	0.78	0.82	13.9
3-x	0.72	0.68	5.4
3-z	0.64	0.65	2.5

**Table 3:** Summary table. Accuracy of the presented methodology.

Case Study	Measured Magnitude	Subcase	Max. Difference (%)
Renzi [26]	Angular distortion	-	0.33
Dye [24]	Cambering distortion	-	8.3
	Angular distortion	-	20
Bhatti [25]	Angular distortion	S355	18.1
		S700	3.9
		S960	8.9
Vetriselvan [27]	Total deformation	Sequence 1-x	34.9
		Sequence 1-z	23.7
		Sequence 2-x	2.2
		Sequence 2-z	13.9
		Sequence 3-x	5.4
		Sequence 3-z	2.5
